# The Effect of an Immersive Virtual Reality Physical Activity Intervention on Anthropometric Variables, Physical Fitness, and Blood Pressure in College Students: A Randomized Controlled Trial

**DOI:** 10.3390/healthcare14040446

**Published:** 2026-02-11

**Authors:** Andrés Godoy-Cumillaf, Paola Fuentes-Merino, Josivaldo de Souza-Lima, Frano Giakoni-Ramírez, Catalina Muñoz-Strale, Maribel Parra-Saldias, Daniel Duclos-Bastias, Claudio Farias-Valenzuela, Eugenio Merellano-Navarro, José Bruneau-Chávez

**Affiliations:** 1 Grupo de Investigación en Educación Física, Salud y Calidad de Vida (EFISAL), Facultad de Educación, Universidad Autónoma de Chile, Santiago 7500915, Chile; paola.fuentes@uautonoma.cl; 2 Facultad de Educación y Ciencias Sociales, Instituto del Deporte y Bienestar, Universidad Andres Bello, Las Condes, Santiago 7550000, Chile; josivaldo.desouza@unab.cl (J.d.S.-L.); frano.giakoni@unab.cl (F.G.-R.); catalina.munoz@unab.cl (C.M.-S.); 3 Departamento de Educación Física, Deporte y Recreación, Universidad de Atacama, Copiapó 1530000, Chile; maribel.parra@uda.cl; 4 iGEO, Escuela de Educación Física, Facultad de Filosofía y Educación, Pontificia Universidad Católica de Valparaíso, Valparaíso 2340025, Chile; daniel.duclos@pucv.cl; 5 METIS Research Lab, Facultad de Negocios y Tecnología, Universidad Alfonso X el Sabio (UAX), 28691 Madrid, Spain; 6 Escuela de Ciencias de la Actividad Física, el Deporte y la Salud, Universidad de Santiago de Chile (USACH), Santiago 9170022, Chile; claudio.farias.v@usach.cl; 7 Department of Physical Activity Sciences, Faculty of Education Sciences, Universidad Católica del Maule, Talca 3530000, Chile; emerellano@ucm.cl; 8 Departamento de Educación Física, Deportes y Recreación, Universidad de la Frontera, Temuco 4811230, Chile; jose.bruneau@ufrontera.cl

**Keywords:** cardiorespiratory fitness, dynamometry, BMI, systolic, diastolic

## Abstract

**Background/Objectives**: University students exhibit high levels of sedentary behavior and low adherence to physical activity recommendations, and immersive virtual reality (IVR) represents an innovative strategy to increase physical activity participation. The aim of this study was to evaluate the effect of a physical activity intervention using IVR on anthropometric variables, physical fitness, and blood pressure in university students. **Methods**: A randomized controlled trial was conducted with 60 participants (30 control, 30 intervention) over 12 weeks. The intervention group performed three weekly exercise sessions using IVR, while the control group maintained their usual activity. BMI, waist and hip circumferences, handgrip strength, cardiorespiratory fitness, and blood pressure were assessed. Baseline characteristics between groups were compared using Student’s *t*-test. The effect of the intervention was analyzed using analysis of covariance adjusted for baseline values. Sensitivity analyses were performed to assess between-group changes, and subgroup analyses were conducted to determine the impact of sex. **Results**: The intervention produced significant improvements in cardiorespiratory fitness (VO_2_ and the 20 m shuttle run test); no significant changes were observed in anthropometric variables, strength, or blood pressure. **Conclusions**: A 12-week intervention with immersive virtual reality-based physical training improves cardiorespiratory fitness in university students, representing a promising tool for health promotion in this population.

## 1. Introduction

Regular engagement in physical activity is widely recognized as a fundamental pillar for health promotion and the prevention of chronic non-communicable diseases, including cardiovascular disease, type 2 diabetes, certain types of cancer, depression, and cognitive impairment across different age groups, including young adults [1,2,3]. Accordingly, evidence indicates that higher levels of physical activity and greater cardiorespiratory fitness are associated with reduced all-cause and cardiovascular mortality, as well as a lower risk of developing metabolic syndrome and other cardiometabolic disorders [4,5,6].

Concurrently, it has been consistently reported that structured exercise programs elicit significant improvements in anthropometric and physical fitness indicators. Interventions lasting 8 to 24 weeks and involving moderate- to vigorous-intensity aerobic exercise, or combined aerobic and resistance training protocols, have been associated with reductions in body mass index (BMI), waist circumference, and central adiposity, together with improvements in blood pressure, lipid profile, and insulin sensitivity [7,8,9]. Moreover, even relatively modest increases in cardiorespiratory fitness are linked to substantial reductions in the risk of all-cause mortality and cardiovascular events [4,5], as well as to enhanced handgrip strength, a functional marker associated with a lower risk of cardiovascular disease, disability, and mortality [10,11]. Collectively, improvements in anthropometric indicators (e.g., BMI and body circumferences) and physical fitness (cardiorespiratory fitness and muscular strength) contribute to a more favorable overall health profile.

In recent years, the integration of technologies such as immersive virtual reality (IVR) into physical activity practice has emerged as an innovative strategy to enhance exercise participation and adherence, particularly among young people. Exercise programs delivered through IVR can elicit effort intensities comparable to those achieved with traditional exercise and functional training modalities [12,13]. Recent trials have demonstrated that video game- and IVR-based interventions lasting 6 to 12 weeks result in significant improvements in cardiorespiratory fitness, as well as reductions in BMI, fat mass, and waist circumference in young and adult populations, including individuals with overweight or obesity [12,13,14]. Furthermore, systematic reviews have reported that IVR use is associated with increased energy expenditure, greater time spent in light- to moderate-intensity physical activity, and improvements in physical and mental health outcomes, including depressive symptoms, perceived stress, and quality of life [15,16]. Specifically in university students, IVR-based exercise has been shown to reduce perceived exertion, increase enjoyment, and enhance intentions to adhere to exercise programs compared with traditional training approaches [17,18].

University students constitute a population of particular interest from a public health perspective. Although they are generally young and apparently healthy, numerous studies have shown that a substantial proportion of university students fail to meet minimum physical activity recommendations and exhibit high levels of sedentary behavior [19,20,21]. Accordingly, meta-analyses indicate that many students spend several hours per day engaged in sedentary activities (e.g., attending lectures, studying, and screen use) and accumulate limited amounts of moderate- to vigorous-intensity physical activity, with these patterns tending to worsen during the transition from secondary to higher education [22,23]. This period has been associated with increases in body weight and BMI, as well as with declines in physical fitness, a phenomenon commonly referred to as the “first-year weight gain” in college students [22,23,24]. Moreover, suboptimal levels of cardiorespiratory fitness and muscular strength have been reported, together with unhealthy lifestyle behaviors (e.g., poor sleep quality, inadequate diet, alcohol consumption, and excessive screen time), collectively constituting a risk profile for the future development of cardiometabolic diseases and mental health disorders [19,20,24,25].

In this context, there is a clear need to implement attractive and accessible interventions that promote regular engagement in physical activity and enhance physical fitness among university students. Immersive virtual reality may represent a promising approach to address this challenge by integrating playful, immersive, and real-time feedback elements that increase motivation and enjoyment during exercise [12,15,17]. However, despite growing evidence suggesting beneficial effects of IVR-based exercise on selected health outcomes, there remains a paucity of controlled, medium-term studies evaluating its impact on anthropometric variables, physical fitness, and blood pressure in the university population. Accordingly, the aim of the present study is to determine the effects of a 12-week physical activity intervention delivered through immersive virtual reality on anthropometric measures, physical fitness, and blood pressure in university students. To this end, a randomized controlled trial was designed. It is hypothesized that IVR-based physical activity will lead to significant improvements in anthropometric and fitness indicators, as well as reductions in blood pressure, thereby providing further evidence supporting immersive virtual reality as an effective tool for health promotion in this population.

## 2. Methodology

### 2.1. Design

A two-arm randomized controlled trial (RCT) was conducted to evaluate the effects of a physical activity intervention delivered through immersive virtual reality on anthropometric variables, physical fitness, and blood pressure in university students. The intervention lasted 12 weeks, with three sessions per week, and was carried out between August and October 2025. The study protocol adhered to the Consolidated Standards of Reporting Trials (CONSORT) guidelines for randomized clinical trials [26].

### 2.2. Ethical Considerations

All procedures were conducted in accordance with the Declaration of Helsinki. The study protocol was approved by the Scientific Ethics Committee of the Universidad Autónoma de Chile (No. 26–24). All participants provided written informed consent prior to enrollment. In addition, the trial was registered at ClinicalTrials.gov (NCT06580769), and the study protocol has been previously published [27].

### 2.3. Participants

The target population consisted of university students from all faculties of a higher education institution in the city of Temuco, Chile. Participants were invited to take part in the study through announcements sent to their institutional email accounts, posts on social media, and informational posters displayed on campus. The inclusion criteria were as follows: (1) enrollment as a university student; (2) age between 18 and 25 years; (3) medical clearance to engage in physical activity; and (4) provision of written informed consent. The exclusion criteria included: (1) the presence of a physical or mental condition precluding participation in physical activity; (2) visual impairment that would prevent the use of immersive virtual reality; and (3) belonging to a special population group (e.g., professional athletes or pregnant women). Prior to enrollment, participants underwent an initial clinical screening based on self-report and medical history to identify diagnosed visual impairments, epilepsy, a history of seizures, photosensitivity, vestibular disorders, recurrent dizziness, or symptoms compatible with cybersickness that could contraindicate or compromise the safe use of immersive virtual reality. All assessments and interventions were conducted using the facilities and equipment of the Physical Education Pedagogy program.

### 2.4. Sample Size Calculation and Randomization

**Figure 1:**
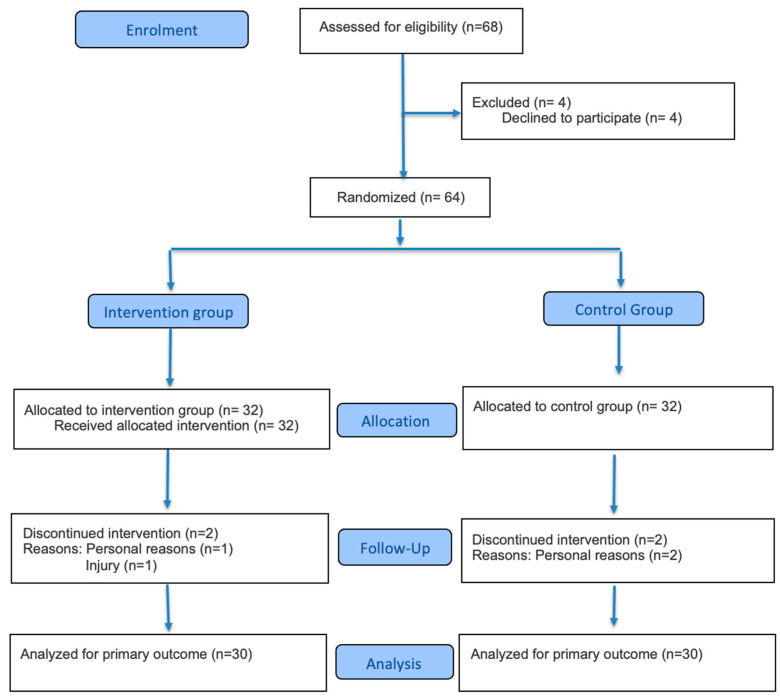
Study design.

The sample size was estimated following the recommendations of Grayling and Watson for the design of clinical trials [28]. Calculations were based on a medium effect size (Cohen’s d = 0.5), a two-sided alpha level of 0.05, and a statistical power of 0.80, using as reference a randomized controlled trial that evaluated changes in health-related variables following an IVR-based exercise intervention [29]. The minimum required sample size was therefore 18 participants. The final sample comprised 60 participants, with 30 allocated to the control group and 30 to the intervention group (Figure 1, flowchart). Participants were assigned to each group (Group 1: control; Group 2: IVR) through simple randomization using an electronic random draw generated with EPIDAT version 4.2 software.

### 2.5. Intervention

#### 2.5.1. Procedures

Once the study sample was established, comprising 64 students from different academic programs (12 from Physical Therapy, 12 from Law, 7 from Psychology, 9 from Accounting, 8 from Chemistry and Pharmacy, 12 from Occupational Therapy, and 4 from Physical Education), all participants were invited to sign the informed consent form. Baseline assessments were then scheduled prior to the start of the intervention. After completion of the 12-week intervention, the same evaluations were conducted; however, only 60 participants were reassessed, as four individuals (two from each group) discontinued the intervention.

The following protocol was applied for the assessment procedures. First, blood pressure was measured, for which participants were required to be in a fasting state. Prior to the measurement, each participant rested for 15 min in the supine position. Anthropometric measurements were then obtained. These assessments were conducted between 8:00 and 9:00 a.m. Afterward, participants were allowed to consume a light meal. Between 10:00 and 11:00 a.m., physical fitness assessments were performed, starting with the handgrip strength test and concluding with the cardiorespiratory endurance test.

The assessors were trained in the measurement protocols and were blinded to group allocation.

Training sessions were scheduled individually for each participant according to their availability. All sessions were conducted in the Virtual Reality Laboratory of the Universidad Autónoma de Chile, Temuco campus.

Participants were instructed to maintain their usual dietary and hydration habits, to avoid major changes in their diet, to attend sessions adequately hydrated, and to hydrate during and after each session. They were asked to refrain from caffeine consumption prior to each session and to avoid engaging in physical activity before the pre- and post-intervention assessments. In addition, participants were instructed not to perform any other type of physical activity during the 12-week intervention period. Those taking medication were required to maintain the same dosage throughout the intervention and to report any changes to the research team. Finally, each participant completed their three weekly training sessions at the same scheduled time each week.

#### 2.5.2. Intervention Group

The program was implemented using a head-mounted virtual reality display (Meta Quest 2, Meta Platforms Inc., Menlo Park, CA, USA) with a screen refresh rate of 120 Hz. The intervention was delivered through the FITXR application (version 3.7.88, developed by FITXR LIMITED, London, UK), which is an exergame that combines punching actions with lateral movements. Before and during the sessions, the headset was individually adjusted for each participant according to their morphological characteristics.

Each session began with a 5 min warm-up at moderate intensity (corresponding to 60–70% of maximal heart rate). The program comprised three progressive levels of increasing complexity according to movement execution speed: beginner, intermediate, and advanced. Each level lasted 10 min, reaching intensities between 80% and 95% of maximal heart rate. Previous studies have indicated that these immersive virtual reality-based routines elicit moderate- to vigorous-intensity exercise responses [30]. The session concluded with a 5 min cool-down phase at 50–60% of maximal heart rate, consisting of mobility and stretching exercises. The total duration of each training session was 40 min.

As safety measures, participants performed the intervention in a clear space of at least 3 m^2^ under continuous supervision. They were instructed to stop the session immediately if they experienced dizziness, nausea, or any other discomfort.

Given the total duration of the sessions (40 min) and the sustained repetition of exercise bouts interspersed with periods of active recovery, the intervention can be classified as high-intensity aerobic training; however, during peak effort periods, a contribution from the anaerobic energy system is also expected.

Moreover, this is consistent with evidence indicating that high-intensity training involves a mixed contribution of both energy systems and provides sufficient stimuli to induce meaningful cardiorespiratory adaptations [31]. Likewise, exercise intensities between 80% and 95% of maximal heart rate elicit a substantial increase in energy expenditure and a marked ventilatory demand, which favor improvements in maximal oxygen uptake and cardiorespiratory efficiency [31].

Finally, the systematic structure of each session, including a progressive warm-up, intervals of high-intensity effort, and active recovery, is designed to promote sustained cardiovascular and metabolic adaptations, in line with the reductions in blood pressure reported in similar high-intensity training programs [32].

All immersive virtual reality sessions were conducted under continuous supervision by a physical education professional in a clear and delimited area. Exercise intensity and participant safety were monitored throughout the sessions, and predefined stopping criteria were applied in the event of discomfort or adverse symptoms.

#### 2.5.3. Control Group

Participants allocated to this group received general information regarding the benefits of physical exercise. Throughout the study period, they were instructed not to initiate any structured physical activity program or to voluntarily increase the duration or intensity of their habitual physical activity; this instruction was reinforced weekly through messages sent to their mobile phones.

#### 2.5.4. Criterion of Adherence to the Intervention

Participants were considered compliant with the intervention if they attended at least 70% of the scheduled training sessions.

### 2.6. Study Variables

#### 2.6.1. Anthropometric

Body weight was measured using a digital scale (Omron, Kyoto, Japan), with participants wearing light clothing. Stature was assessed with a stadiometer (Dry), with participants barefoot, standing upright, and at the end of a normal inspiration. Both measurements were obtained in duplicate, and the mean value was used to calculate body mass index (BMI, kg/m^2^). Waist circumference was measured at the midpoint between the lower margin of the last rib and the upper border of the iliac crest, whereas hip circumference was measured at the level of the greatest gluteal protuberance, corresponding to the level of the pubic symphysis. Both circumferences were measured in duplicate using a flexible measuring tape (Holway, San Jose, CA, USA), and the mean of the two measurements was used for subsequent statistical analyses.

#### 2.6.2. Physical Fitness

Handgrip strength was assessed using a digital dynamometer (Takei 5401, Takei Scientific Instruments Co., Ltd. (Niigata, Japan)). Participants were instructed to squeeze the device with maximal effort for approximately two seconds, without making contact with any other part of the body. Two trials were performed with each hand, and the mean value was recorded for analysis.

Cardiorespiratory fitness was evaluated using the 20 m shuttle run test [33]. Participants ran back and forth between two lines separated by 20 m, starting at an initial speed of 8.5 km·h^−1^, which increased by 0.5 km·h^−1^ each minute. A pre-recorded audio signal paced the running speed, and participants were required to reach the line at each beep. The test was terminated when the participant failed to reach the line on two consecutive occasions in time with the audio signal. The final completed stage was recorded and used to estimate maximal oxygen uptake (VO_2_max) according to the established prediction equation [33].

#### 2.6.3. Blood Pressure

The patient was evaluated twice by means of an Omrom monitor (Omrom healthcare OK Ltd., Milton Keynes, UK), while the evaluated person was seated, with the arm semi-flexed at the level of the heart.

#### 2.6.4. Exercise Intensity

By performing a maximal cardiorespiratory exercise test, which was supervised by a cardiologist, the intensity of the exercise was determined. A Monark LC7TT (Monark Exercise AB, Vansbro, Sweden) exercise bike and a heart rate monitor Polar H10 (Polar Electro Oy, Kempele, Finland)were used. It began with 5 min of unloaded warm-up; then, every 2 min the intensity was increased by 25 W, maintaining a cadence of 60 rpm. The test was terminated when the person being evaluated could not maintain the requested cadence.

### 2.7. Statistical Analysis

Baseline characteristics between groups were compared using Student’s *t*-test for independent samples. The effect of the intervention was analyzed by analysis of covariance (ANCOVA), with the post-intervention value as the dependent variable, group (intervention vs. control) as the fixed factor, and the baseline value of the corresponding variable as the covariate. The group effect coefficient (β), its 95% confidence interval, and the *p* value were reported. To control for multiple-comparison error across outcomes, *p* values from the ANCOVA were adjusted using the Holm–Bonferroni method; additionally, Benjamini–Hochberg false discovery rate (FDR) adjustment was reported as a complementary analysis. As a sensitivity analysis, between-group differences in change scores (Δ = post − pre) were compared using Welch’s *t*-test for independent samples, and effect sizes were estimated using Cohen’s d [34]. Statistical significance was set at α = 0.05 (two-tailed), with primary inferences based on the adjusted *p* values. Additionally, subgroup analyses were conducted to explore potential effect modification by sex and by baseline physical fitness. Baseline fitness was operationalized using a composite index derived from baseline cardiorespiratory endurance measures (mean of z-scores for baseline VO_2_ and baseline 20 m shuttle run performance) and categorized into tertiles (low, medium, and high). Effect modification was assessed using ANCOVA models that included the subgroup factor and the Group × Subgroup interaction term (model: post = group + baseline + subgroup + group × subgroup). The interaction coefficient (β), its 95% confidence interval, and the corresponding *p* value were reported. All analyses were performed using SPSS version 29.

## 3. Results

Table 1 presents the baseline values for the total sample and stratified by intervention and control groups. Both groups exhibited comparable baseline characteristics.

Table 2 presents the post-intervention values for the control and intervention groups, as well as the group effect estimated by ANCOVA adjusted for the baseline value of each variable. After adjustment for multiple comparisons, statistically significant between-group differences were observed only for the indicators of cardiorespiratory capacity.

Specifically, the intervention group exhibited superior end-of-follow-up performance in the 20 m shuttle run test, with a positive group effect (β = 0.736; 95% CI, 0.515 to 0.957; *p* < 0.001), which remained significant after correction for multiple comparisons (Holm-adjusted *p* < 0.001; FDR-adjusted *p* < 0.001). A significant increase in VO_2_ was also observed in the intervention group (β = 2.044 mL·kg^−1^·min^−1^; 95% CI, 1.337 to 2.752; *p* < 0.001), likewise robust after adjustment (Holm-adjusted *p* < 0.001; FDR-adjusted *p* < 0.001). In contrast, no between-group differences were detected after baseline adjustment for body weight, BMI, waist and hip circumferences, handgrip strength (right and left), or blood pressure (systolic and diastolic).

Supplementary Table S1 presents the sensitivity analysis comparing between-group changes (Δ = post − pre) and effect sizes. The intervention group showed greater improvements in the 20 m shuttle run test (Δ = 1.00 ± 0.37 vs. 0.27 ± 0.45; *p* < 0.001; d = 1.78) and in VO_2_ (Δ = 3.00 ± 1.11 vs. 0.80 ± 1.35 mL·kg^−1^·min^−1^; *p* < 0.001; d = 1.78), both with large effect sizes. No significant between-group differences in change were observed for the remaining variables. Subgroup analyses were conducted to examine the impact of sex on the observed outcomes, including the physical fitness measures. Supplementary Table S2 presents baseline characteristics stratified by sex. Baseline differences between males and females were observed in cardiorespiratory fitness, with higher VO_2_ values and superior performance in the 20 m shuttle run test in men compared with women. Baseline systolic and diastolic blood pressure also differed by sex. Subgroup analyses were conducted using interaction terms in ANCOVA models adjusted for the baseline value of each outcome (Supplementary Table S3). For VO_2_, evidence of effect modification by sex was observed. In contrast, no evidence of effect modification by baseline physical fitness categorized into tertiles was found. For the 20 m shuttle run test, the interaction with sex was marginal, and no interaction by baseline physical fitness was detected.

## 4. Discussion

The aim of this study was to determine the effects of a 12-week immersive virtual reality-based physical activity intervention on anthropometric variables, physical fitness, and blood pressure in university students using a randomized controlled trial design. The main findings indicate that the intervention elicited a marked improvement in cardiorespiratory fitness (VO_2_) in the intervention group.

The most consistent and pronounced outcome was the increase in VO_2_ in the intervention group, with a large effect size. This finding is consistent with evidence indicating that moderate- to vigorous-intensity exercise programs lasting at least 12 weeks can significantly improve cardiorespiratory fitness in young adults and university populations [1,3,35]. Moreover, even modest increases in VO_2_ have been associated with substantial reductions in the risk of all-cause and cardiovascular mortality [4,36]. Therefore, the magnitude of the change observed in the present study suggests a clinically relevant cardiometabolic benefit, even in an initially young and largely healthy population.

Regarding anthropometric variables, BMI and waist and hip circumferences showed non-significant reductions. This pattern is consistent with studies reporting modest initial changes in body weight and BMI following 12–16-week interventions, whereas more pronounced changes in body circumferences and body composition typically require longer intervention periods, greater exercise volumes, or programs combined with nutritional strategies [6,8,37]. As the present intervention focused exclusively on physical activity and did not include a dietary component, the observed decreases in BMI can be considered favorable; however, a longer duration may be necessary to achieve statistically significant improvements in the other anthropometric outcomes.

In contrast, handgrip strength did not show significant changes, a finding that has also been reported in interventions with similar characteristics [38]. The absence of significant improvements in strength may be explained by the nature of the training stimulus applied. The intervention primarily targeted the cardiorespiratory component and involved dynamic movements with low external mechanical load, which likely did not generate sufficient muscular tension to elicit strength adaptations. This result is consistent with the principle of training specificity. Current evidence indicates that improvements in handgrip strength and overall muscular strength require resistance training protocols specifically designed for these outcomes, characterized by moderate- to high-intensity loads and progressive overload [12,39]. Therefore, it is not unexpected that an intervention predominantly focused on cardiorespiratory fitness did not produce significant changes in muscular strength.

Subgroup analyses suggest that sex may act as an effect modifier of the intervention on cardiorespiratory fitness. Specifically, the Group × Sex interaction was significant for VO_2_, which is consistent with previous research documenting sex-related differences in training responses, mediated by physiological and endocrine factors, as well as by differences in baseline fitness levels, body composition, and internal exercise load for a given training prescription [40,41,42]. In contrast, no effect modification by baseline physical fitness was observed, indicating that, at least in this sample, the intervention exerted a relatively consistent effect across initial fitness levels. This finding is also in line with studies showing that improvements are more strongly influenced by adherence and the actual exercise dose completed than by baseline fitness status [4,41].

The findings of this study are consistent with the emerging evidence on the effects of virtual reality-mediated exercise, which has shown that active video games and immersive virtual reality can elicit physiological responses comparable to those of light- to moderate-intensity aerobic exercise and, in some cases, reach vigorous intensities, thereby increasing energy expenditure and time spent in physical activity [13,30,43]. In randomized controlled trials involving young adults and overweight populations, 6- to 12-week virtual reality-based interventions have demonstrated improvements in VO_2_ and modest reductions in BMI and body fat percentage [15,16]. Accordingly, our results showing a marked enhancement in cardiorespiratory fitness and a moderate decrease in BMI agree with these previous reports.

With respect to the population studied, university students are characterized by low compliance with international physical activity recommendations and by spending several hours per day in sedentary behaviors, which are associated with poorer mental health, higher BMI, and lower cardiorespiratory fitness [18,19,20,24]. Therefore, our findings provide evidence that may contribute to improving their health, not only in the variables assessed in this study but also in others. Indeed, studies in university students using immersive virtual reality to promote physical activity have reported improvements in mental health indicators (e.g., reductions in anxiety and depressive symptoms, greater perceived well-being) and increased enjoyment of exercise, which may translate into better medium-term adherence [15,25].

The strengths of this study include the use of a randomized controlled design, the duration of the intervention (12 weeks), and the assessment of a broad set of health-related variables. In addition, the implementation of a supervised immersive virtual reality-based intervention provides novel evidence within a real-world university student context.

Among the limitations of this study is the lack of control over potential confounding variables, such as dietary intake, levels of physical activity outside the intervention, sleep patterns, and psychosocial factors (e.g., motivation or stress), as well as the absence of objective or self-reported assessment of physical activity levels in the control group. These factors may have influenced the observed responses. Future studies should consider assessing and controlling for these variables to strengthen the interpretation of the effects of immersive virtual reality-based exercise interventions.

Future research should explore interventions with a greater training volume or multimodal approaches, such as incorporating specific muscular strength blocks within immersive virtual reality environments or combining them with traditional resistance training to induce neuromuscular adaptations. Extending the duration of the intervention may also help to determine whether longer exposure leads to additional improvements in anthropometric and fitness outcomes. Moreover, future studies should include comprehensive assessments of lifestyle-related health behaviors, including physical activity levels, dietary patterns, sleep quality, and psychosocial factors. Given that obesity is an independent cardiovascular risk factor and that some of these behaviors may be less prevalent among individuals with overweight or obesity, this integrative approach would allow a more precise understanding of how lifestyle behaviors and intervention characteristics interact to modulate cardiovascular risk profiles in the university population.

## 5. Conclusions

This study shows that a 12-week physical activity intervention delivered via IVR in university students is effective in producing a significant improvement in cardiorespiratory fitness compared with a control group that did not participate in this type of program. These findings indicate that IVR may be a valid tool for promoting improvements in health-related components in the university population.

Our findings provide evidence supporting the incorporation of exercise programs using IVR as a health promotion strategy in the university setting, particularly in light of the high prevalence of sedentary behavior and low levels of physical activity in this population.

## Figures and Tables

**Table 1:** Baseline characteristics of the sample.

Characteristic	Total n = 60	Intervention Group n = 30	Control Group n = 30	*p*
Female n (%)	32 (53.3)	16 (53.3)	16 (53.3)	-
Male n (%)	28 (46.7)	14 (46.7)	14 (46.7)	-
Age (years)	21.32 ± 0.82	21.38 ± 1.22	21.78 ± 1.33	0.853
Body mass (kg)	170.25 ± 8.26	170.86 ± 6.7	169.64 ± 9.64	0.571
Height (cm)	76.88 ± 18.1	76.39 ± 16.87	77.37 ± 19.52	0.836
BMI (kg/m^2^)	26.3 ± 4.59	25.95 ± 4.18	26.65 ± 5.01	0.557
Waist (cm)	81.85 ± 12.43	82.29 ± 11.45	81.4 ± 13.52	0.783
Hip (cm)	100.54 ± 7.69	100.72 ± 7.86	100.36 ± 7.65	0.858
Systolic BP (mm/hg)	111.63 ± 19.01	111.33 ± 21.15	111.93 ± 16.97	0.904
Diastolic BP (mm/hg)	74.63 ± 13.89	78.67 ± 14.79	70.6 ± 11.83	0.053
Right hand grip strength (kg)	38.05 ± 10.08	40.35 ± 9.83	35.75 ± 9.96	0.077
Left hand grip strength (kg)	36.48 ± 12.68	38.41 ± 12.43	34.54 ± 12.84	0.240
20 m shuttle (stage *)	4.8 ± 2.04	5.27 ± 2.3	4.33 ± 1.65	0.076
VO_2_ (mL/kg/min)	33.7 ± 6.37	36.4 ± 6.91	31.0 ± 4.44	0.070

* 1 stage = 1 min. BP: Blood pressure.

**Table 2:** Pre- and post-differences in control and intervention groups.

	Control Group Post (n = 30)	Intervention Group Post (n = 30)	β Group	95% IC Inf	95% IC Sup	*P* ^1^	*P* ^2^	*P* ^3^
Body mass (kg)	76.79 ± 18.64	75.15 ± 15.68	−0.711	−1.448	0.025	0.058	0.4546	0.144
BMI (kg/m^2^)	26.47 ± 4.79	25.55 ± 3.88	−0.257	−0.521	0.008	0.056	0.4546	0.144
Waist (cm)	81.4 ± 13.24	82.09 ± 10.97	−0.169	−0.811	0.473	0.599	1	0.749
Hip (cm)	100.07 ± 6.63	100.2 ± 6.88	−0.17	−1.313	0.974	0.767	1	0.767
Systolic BP (mm/hg)	115.53 ± 8.41	114.07 ± 12.54	−1.307	−6.197	3.584	0.594	1	0.752
Diastolic BP (mm/hg)	67.87 ± 5.52	71.13 ± 10.65	1.579	−2.789	5.948	0.472	1	0.60
Right hand grip strength (kg)	36.23 ± 10.63	40.56 ± 12.08	−0.511	−2.957	1.935	0.677	1	0.749
Left hand grip strength (kg)	36.28 ± 12.12	38.45 ± 9.5	−0.985	−2.876	0.907	0.301	1	0.749
20 m shuttle (stage *)	4.6 ± 1.65	6.27 ± 2.36	0.736	0.515	0.957	**0.000**	**0.000**	**0.000**
VO_2_ (mL/kg/min)	31.8 ± 4.92	39.4 ± 7.09	2.044	1.337	2.752	**0.000**	**0.000**	**0.000**

* 1 stage = 1 min. The values in bold indicate a statistical significance of *p* < 0.01. BP: Blood pressure. *P*^1^: ANCOVA. *P*^2^: adjusted by Holm–Bonferroni. *P*^3^: Adjusted by Benjamini–Hochberg.

## Data Availability

The data presented in this study are available on request from the corresponding author. The data are not publicly available due to ethical standards.

## Supplementary Materials

The following supporting information can be downloaded at: https://www.mdpi.com/article/10.3390/healthcare14040446/s1. Table S1. Between-group sensitivity analysis. Table S2. Baseline characteristics of the study participants by sex. Table S3. Subgroup analysis.

## Abbreviations

The following abbreviations are used in this manuscript:

BMI	Body mass index
IVR	Immersive virtual reality
VO_2_	Oxygen uptake
RCT	Randomized control trial
