# Supplementary material for: The Effect of an Immersive Virtual Reality Physical Activity Intervention on Anthropometric Variables, Physical Fitness, and Blood Pressure in College Students: A Randomized Controlled Trial
Source: Healthcare (Basel). 2026 Feb 11;14(4):446. doi: 10.3390/healthcare14040446 (PMC12941052; doi:10.3390/healthcare14040446)
Supplement: Supplementary file 1 [file healthcare-14-00446-s001.zip › healthcare-4103560-supplementary.pdf]

Table S1. Between-group sensitivity analysis

	Δ Intervention Group (media ± SD)	Δ Control Group (media ± SD)	p	d
Body mass (kg)	-1.24 ± 2.18	-0.59 ± 1.26	0.161	-0.368
BMI (kg/m^2)	-0.39 ± 0.72	-0.18 ± 0.39	0.162	-0.367
Waist (cm)	-0.2 ± 1.79	0.0 ± 0.44	0.557	-0.153
Hip (cm)	-0.52 ± 3.24	-0.29 ± 1.69	0.728	-0.09
Systolic BP (mm/hg)	2.73 ± 19.88	3.6 ± 13.27	0.843	-0.051
Diastolic BP (mm/hg)	-7.53 ± 15.26	-2.73 ± 10.94	0.167	-0.362
Right hand grip strength (kg)	0.21 ± 5.73	0.48 ± 3.06	0.823	-0.058
Left hand grip strength (kg)	0.03 ± 5.04	1.74 ± 3.37	0.129	-0.398
20 m shuttle (stage)	1.0 ± 0.37	0.27 ± 0.45	**<0.001**	1.778
VO2 (ml/kg/min)	3.0 ± 1.11	0.8 ± 1.35	**<0.001**	1.778

The values in bold indicate a statistical significance of p < 0,05. BP: Blood pressure.

Table S2. Baseline characteristics of the study participants by sex

Variable basal	Women (media ± SD)	Men (media ± SD)	p
VO2 (ml/kg/min)	29.6 ± 3.21	36.07 ± 6.56	**<0.001**
20 m shuttle (stage)	3.73 ± 1.58	5.42 ± 2.04	**<0.001**
Body mass (kg)	71.42 ± 16.61	80.05 ± 18.37	0.068
BMI (kg/m^2)	24.84 ± 4.13	27.14 ± 4.68	0.054
Waist (cm)	79.27 ± 10.12	83.34 ± 13.49	0.192
Hip (cm)	98.63 ± 5.68	101.65 ± 8.52	0.106
Systolic BP (mm/hg)	95.73 ± 22.48	120.84 ± 7.22	**<0.001**
Diastolic BP (mm/hg)	82.09 ± 17.85	70.32 ± 8.62	**0.007**

The values in bold indicate a statistical significance of p < 0,05. BP: Blood pressure.

Table S3. Subgroup analysis

			IC95%		
**Outcome**	**Modificatory**	**β**	**LL**	**UL**	**p**
VO2 (ml/kg/min)	Sex	1,993	0.58	3.405	**0.007**
VO2 (ml/kg/min)	Baseline physical fitness (tertiles)	0,101	-0.662	0.863	0.792
20 m shuttle (stage)	Sex	0,487	-0.028	1.001	0.063
20 m shuttle (stage)	Baseline physical fitness (tertiles)	-0,143	-0.419	0.132	0.302
Body mass (kg)	Sex	-0,556	-2.203	1.092	0.502
Body mass (kg)	Baseline physical fitness (tertiles)	0,634	-0.374	1.641	0.213
BMI (kg/m^2)	Sex	-0,035	-0.618	0.549	0.906
BMI (kg/m^2)	Baseline physical fitness (tertiles)	0,128	-0.238	0.493	0.487

The values in bold indicate a statistical significance of p < 0,05. LL, lower limit of the 95% confidence interval; UL, upper limit of the 95% confidence interval.
